# Lifetime Modulation of the Pain System *via* Neuroimmune and Neuroendocrine Interactions

**DOI:** 10.3389/fimmu.2017.00276

**Published:** 2017-03-13

**Authors:** Ihssane Zouikr, Bianka Karshikoff

**Affiliations:** ^1^Laboratory for Molecular Mechanisms of Thalamus Development, RIKEN BSI, Wako, Japan; ^2^Department of Clinical Neuroscience, Division for Psychology, Karolinska Institutet, Solna, Sweden; ^3^Stress Research Institute, Stockholm University, Stockholm, Sweden

**Keywords:** psychoneuroimmunology, lipopolysaccharide, inflammation, pain, neuroimmunology, neuroendocrinology, hypothalamo–pituitary–adrenal axis, stress

## Abstract

Chronic pain is a debilitating condition that still is challenging both clinicians and researchers. Despite intense research, it is still not clear why some individuals develop chronic pain while others do not or how to heal this disease. In this review, we argue for a multisystem approach to understand chronic pain. Pain is not only to be viewed simply as a result of aberrant neuronal activity but also as a result of adverse early-life experiences that impact an individual’s endocrine, immune, and nervous systems and changes which in turn program the pain system. First, we give an overview of the ontogeny of the central nervous system, endocrine, and immune systems and their windows of vulnerability. Thereafter, we summarize human and animal findings from our laboratories and others that point to an important role of the endocrine and immune systems in modulating pain sensitivity. Taking “early-life history” into account, together with the past and current immunological and endocrine status of chronic pain patients, is a necessary step to understand chronic pain pathophysiology and assist clinicians in tailoring the best therapeutic approach.

## Introduction

The pain system is modulated by neuroimmune and neuroendocrine mechanisms from embryonic development throughout life. Unlike the traditional reductionist view that posits that pain is solely due to aberrant spinal and supraspinal neuronal activity, we now understand pain in the context of a complex multisystem comprising well-organized interactions between neuroendocrine and neuroimmune systems (1). The changes in the nervous system induced by the immune system and the endocrine system are of both structural and functional character and are a part of the normal, adaptive development of the pain system. However, an adaptation that is advantageous in one situation may pose a risk factor in another. Exposure to a wide range of stressors, from physical injury (such as incision) to infection and inflammation [as induced by, e.g., lipopolysaccharide (LPS)], activates the hypothalamo–pituitary–adrenal (HPA) axis as well as peripheral and central immune responses and reorganizes the sensitivity of the pain system (2–5). The HPA axis and neuroimmune activation are of importance in determining long-term pathological states such as chronic pain.

Treating chronic pain is complicated by the wide individual differences in symptoms and treatment response. Chronic pain is also associated with a high incidence of psychiatric comorbidity (6) and is often present with other primary diagnoses, such as inflammatory disease. Furthermore, stress is often directly targeted in behavioral treatment strategies for chronic pain (7), as part of an integrated treatment approach (8, 9). In this study, we explore some of the biological mechanisms that may form the foundations of the complexity seen in clinical pain.

This review focuses on some of the mechanisms involved in the maturation of the nervous system, which define the function of the pain system later in life. We highlight the importance of neuroimmune and neuroendocrine interactions very early in life in the programming of the pain system. We also discuss how the immune system and the endocrine system continue to modulate pain processing throughout life and about the significance of these interactions for chronic pain.

## Ontogeny of the Central Nervous System (CNS) During the Prenatal and Postnatal Period

Neuronal circuits are forged by sensory experiences. Exposure to environmental stressors during a critical period of brain ontogeny, when neuronal circuits are particularly sensitive to modification by experience, can have long-term consequences on neural circuits, ultimately affecting behavior (10). Although our genetic makeup determines much of the structure and function of the nervous system, the environment where the individual is born, as well as the environmental conditions that will accompany the individual throughout his/her life, plays a crucial role in tailoring the neuronal properties. The postnatal developing nervous system responds to the external world to shape its neural circuits in order to subserve a particular function (i.e., vision, auditory, touch, etc.). In normal conditions (i.e., in the absence of any adverse events), non-stressful early experience specifies a neural trajectory to the best possible circuits of connectivity. In other words, non-efficient connections are eliminated and those that are functionally stable remain. However, if exposed to stress—whether it is of physical, physiological, psychological, or viral/bacterial nature—during a time when the brain is still undergoing fine-tuned maturation, the process of synaptic plasticity, or synaptic tuning can go seriously wrong, affecting the behavioral outcome.

### Early Development of the Human Brain

During the prenatal period, the brain produces approximately 250,000 cells per minute (11). Neuronal migration occurs between gestational week (GW) 8 and 16 forming the subventricular zone (SVZ) (12). Around GW 16, neurons reach their final target and begin to form connections among brain regions (13). Synapse formation in both the auditory and prefrontal cortices begins around GW 27 (14). During the beginning of the third trimester, synaptogenesis occurs with a rate of approximately 40,000 synapses per minute (15). Subsequently, myelination as well as proliferation and differentiation of oligodendrocytes (cells that produce myelin) take place. After birth, the size of the brain continues to increase dramatically, with intense metabolic changes associated with synapse formation and axonal growth during the first 3 months of postnatal life (16). The way the complex human brain develops and matures is through a significant increase in volume due to overproduction of synapses, myelination, and connections during infancy, followed by the elimination of less efficient synapses *via* pruning (17). Most importantly, the developmental trajectory of the neocortex is different depending on brain regions. For instance, the primary visual cortex undergoes significant maturation during the first 3 months of life, whereas the primary auditory cortex continues to mature over the first 3 years of life (18). The bilateral thalamic connectivity to the prefrontal cortex (PFC) is increased gradually from childhood to late teens (19), and synaptic pruning in the PFC continues to occur in mid-adolescence (14). The relatively late maturation of thalamo–PFC synaptic connections implies that key connections involved in complex cognitive functions, including pain, are still undergoing fine-tuned maturation in early postnatal life. Consequently, exposure to stressful events such as viral/bacterial infections during postnatal life is likely to be able to alter key neural circuits involved in pain processing. This may lead to altered pain responses later in life. At present, there is a paucity of research tackling this question, and further studies investigating the impact of early-life stress on neural circuits involved in pain processing are needed.

### What Animal Models Reveal about Neurogenesis and Synaptic Plasticity

The traditional dogma posits that the postnatal brain (including adult brain) possesses a fixed number of neurons that are generated from birth and that no neurogenesis or synaptic plasticity is possible in the adult brain (20). However, it is now clear that neurogenesis and synaptic plasticity continue to occur in the adult brain, although at a lower rate. Findings from studies that used standard neuronal markers, such as NeuN and bromodeoxyuridine (BrdU), have detected postnatal neurogenesis both in primates and rodents. NeuN^+^/BrdU^+^ cells were detected particularly in two regions: the SVZ–olfactory bulb and the subgranular zone (SGZ)–hippocampal granule cell layer (21–25). Regarding synaptic plasticity in the adult brain, pioneer studies by Merzenich et al. demonstrated that amputation of one finger in adult monkeys resulted in deafference of the devoted territory within the somatosensory cortex and that this region compensated by receiving inputs from neighboring fingers (26). Later on, Robertson and Irvine showed that similar compensatory mechanisms and cortical rearrangement occurred in the auditory cortex following lesion of the cochlea (27).

In rats, PFC neural circuits undergo significant changes during the perinatal period. The myelination of the medial PFC (mPFC) is very low at P7, increases gradually over the period P21–P50, and reaches peak level at P90 (28). The ontogenic development of the PFC implies that this region, which plays a critical role in cognitive functioning and pain processing (29), is particularly susceptible to environmental stimuli during the neonatal period. Consequently, exposure to stressful events during this period is likely to alter the neural circuits within the PFC—and consequently pain processing later in life. Indeed, sensory, painful, or stressful experience has been shown to change the dendritic and spine morphology in this area. A combination of prenatal stress (E14–E21) and maternal separation (P2–P21) resulted in increased c-Fos expression in the mPFC and reduced dendritic length and dendritic spines of mPFC neurons (30). A recent study has found that pyramidal neurons from the mPFC of spared nerve injury (SNI) rats are characterized by longer basal dendrites and increased spine density compared to sham-operated animals (29). Electrophysiological recording of mPFC pyramidal neurons from SNI rats revealed increased NMDA/AMPA ratio in currents evoked by stimulation of layer 5 (29). However, convincing data linking directly altered PFC neural circuits following early-life stress to future pain responses are still lacking.

Taken together, these data suggest that the perinatal, up to and including the early childhood period, is a time of high plasticity for the brain and adverse events occurring during this critical period of cellular proliferation, differentiation, and maturation can interfere with the normal developmental trajectory of the brain, resulting in structural and/or functional changes in cells, tissues, or organ systems. These changes are proposed to potentially lead to increased susceptibility to neurodevelopmental disorders in later life (31–33) and may also be critical for determining adult pain responses and potentially the susceptibility to develop chronic pain.

### Early-Life Development of the Pain System

One of the neuronal systems that undergo significant malleability during the perinatal period is the nociceptive system. For instance, at embryonic day (E) 15–17 myelinated A fibers are the first to penetrate the spinal lumbar cord before the subsequent projection of C fibers into the substantia gelatinosa (lamina II, superficial dorsal horn that contains nociceptive-specific neurons) at E19 (34). During the neonatal period, lamina II is innervated by both A- and C fibers. During the first 3 weeks of postnatal age, a withdrawal of A fiber primary afferents into deeper laminae is noticed, and C fibers exclusively innervate lamina II at the adult stage (35). This developmental pattern of nociceptive fibers is of particular relevance to the concept that early-life insults are able to alter the neuroanatomical components of nociception (including nociceptive fibers), leading to altered pain responses later in life. For instance, skin wound during the neonatal period is associated with hyperinnervation of the wounded area by both Aδ and C fibers (36, 37). This hyperinnervation of nociceptive fibers can lead to peripheral sensitization and increased pain sensitivity (i.e., hyperalgesia). Despite the apparent lack of maturity of the nociceptive system, overall, younger animals are markedly more sensitive to noxious stimuli than their adult counterparts (38). Their behavioral output may, however, differ from adult animals. The withdrawal threshold from heat stimuli is lower in young animals compared to adults, and neonatal rats are significantly more (i.e., 10-fold higher) sensitive to formalin injection than preadolescent rats who require higher formalin doses to elicit the formalin-induced behavioral responses (38). For example, until P10, injection of formalin into the hind paw elicits predominantly non-specific whole body movement (i.e., jerking), whereas the formalin-induced specific behaviors such as hind paw shaking, flexion, and licking appears only after P10 (39). Of particular interest, recent studies predominantly from Hathway et al. elegantly demonstrated that the descending inhibitory control of spinal nociceptive reflexes from the periaqueductal gray (PAG) to rostroventral medullar (RVM) in rats undergoes an important developmental switch from facilitatory in young rats to inhibitory in adult rats (40–42). This developmental switch was found to be driven by opioid actions on RVM, as microinjection of the μ-opioid agonist [d-Ala2, N-MePh4, glycol]-enkephalin (DAMGO) into RVM facilitates spinal nociceptive reflexes in preadolescent rats (P21), but elicited antinociceptive actions in adult rats (42), and similar response pattern has also been recently shown to occur at the PAG level (41).

Overall, a number of neural systems, including those involved in pain modulation, are characterized by significant malleability illustrated by major structural and functional rearrangements in neural circuits following insult. This injury-induced plasticity renders the nociceptive system more vulnerable to future challenges. Why certain patients develop chronic pain while others do not might in fact result from different early-life experiences in these patients, which may have programmed the pain system differently later in life. Therefore, taking “early-life history” into account is a necessary step to understand chronic pain pathophysiology and developing individual-based therapeutic strategies (43).

## Ontogeny of the HPA Axis During the Perinatal Period

### Prenatal Development

The experience of stress, from an evolutionary perspective, is very important in promoting survival of an organism. A fundamental system that is subjected to programming by early-life events is the neuroendocrine axis that mediates the stress response, the HPA axis (44, 45). Activation of this system starts with the recruitment of neurons within the paraventricular nucleus of the hypothalamus (PVN), and the end product is the release from the adrenal cortex of corticosterone for rodents or cortisol for humans, *via* the release of corticotropin-releasing hormone (CRH) and adrenocorticotropic hormone (ACTH) [the HPA axis has been extensively reviewed elsewhere, please see Ref. (46)]. During pregnancy, there is an increase in CRH production in the placenta and fetal membranes. The gradual increase in maternal HPA axis activity during this period leads to maternal hypercortisolemia (47, 48). The fetus has much lower levels of glucocorticoids than its mother although endogenous glucocorticoids can cross the placenta easily. A total of 10–20% of cortisol present in the amniotic liquid is from maternal origin, while the remaining 80–90% gets converted into inactive cortisone by an enzyme, 11β-HSD2, to protect the fetus’s brain from excess glucocorticoids, which can be neurotoxic (49). During the third trimester, fetal 11β-HSD2 levels decrease, and the fetus is exposed to high levels of CRH and cortisol. This rise in CRH and cortisol levels is thought to play an important role in the maturation of organs and preparation of the fetus to the *ex utero* environment (50). The hippocampus plays a key role in regulating homeostatic levels of glucocorticoids under conditions of stress, and CRF has been shown to modulate the electrical activity of hippocampal neurons (51). Glucocorticoid receptor (GR) mRNAs were detected in human fetal hippocampus at 24 GWs (52). Additionally, fibers expressing CRH have been detected in humans by GW16 (53), and the release of CRH into the pituitary has been reported to occur at GW11.5 (54). At the pituitary, a basic adenohypophysis can be detected at GW6 (55), and by GW8 the pituitary reaches mature stage and can release ACTH (56).

In rodents, GR mRNA can be detected in the telencephalon as early as E12.5 with high expression seen in the anterior hypothalamus, pons, spinal cord, and pituitary gland (57). At E14.5, the expression of GR mRNA significantly increases in the ventral spinal cord and the thalamus and undergoes a moderate decrease in these regions by E15.5. An increase in GR mRNA levels is observed at the same time point in other regions including the neocortex, cerebellum, and basal ganglia (57). In the PVN, GR mRNA can be visible at E16, although it is not clear whether this PVN GR is functional at this stage (58). During the late gestation (E17–E19), GR mRNA is localized in the hippocampus, thalamus, and the amygdala (58). Mineralocorticoid receptors (MR) ontogenetic expression, however, follows a different pattern in rodents. MR mRNA expression cannot be detected before E15.5 when a moderate expression is observed in pituitary gland, brain stem, tegmentum, and neuroepithelium of the septum and pallidum (57). MR mRNA expression is first seen in the hypothalamus at E17.5 and by E19.5 there is a dramatic increase in MR mRNA expression in the hippocampus, septum, anterior hypothalamus, PAG area, and brainstem neuroepithelium (57). Regarding the ontogeny of 11β-HSD2 mRNA (encoding an enzyme that converts corticosterone into its inactive form) in rodents, the expression of 11β-HSD2 mRNA is observed at E11.5 on hippocampal and subicular regions, neocortex, septum, and posterior hypothalamic area. At E14.5, the expression intensity of 11β-HSD2 mRNA starts to decline in the neocortex, pallidal area, and spinal cord, and by E15.5 11β-HSD2 mRNA is restricted to the thalamus, midbrain, striatum, cerebellum, hypothalamus, medulla, and pallidum (57).

### Postnatal Modulation

There is a particular period called “the stress hyporesponsive period” (SHRP) from P4 to P14 in rats and from P2 to P12 in mice during which corticosterone levels, as well as ACTH, are maintained at low levels even in the presence of mild stress (59). Although, it is generally accepted that pups do not respond to stress with an elevated HPA axis activity during the SHRP period, it has been reported that 12 day-old pups that were separated from their mothers for 24 h with no access to food or water showed a significant increase in both basal and stress-induced corticosterone and ACTH secretion (59, 60). These results indicate that the HPA axis is particularly sensitive to maternal care even during the SHRP. During this period, high expression of CRH is observed in the PVN, whereas hippocampal GR expression is low at birth and increases gradually during the SHRP (61). *In situ* hybridization studies in marmoset showed that the ontogenetic profile of MR and GR is different during the postnatal period. Although GR mRNA expression in the dentate gyrus is higher in 4–6 week-old marmoset than in neonates (P1–P2), juveniles (4–5 months), and adult (3–6 years), MR mRNA expression was developmentally consistent in the hippocampus and PVN throughout life (62).

Although we need to proceed with caution when extrapolating from animal studies to humans, the development of the brain in terms of synapse formation and brain growth rate in a P6 rat is relatively equivalent to 38–40 weeks of gestation in humans (63, 64). For obvious ethical and methodological reasons, human data regarding the ontogenetic development of HPA axis are lacking. However, we can conclude from the abovementioned animal data that the prenatal period together with the first 2 weeks of postnatal life constitute a window of significant plasticity for the neuroendocrine system. Homeostasis of the neuroendocrine function, and consequently any physiological system that is under the influence of this system (e.g., pain), is needed for normal neuroendocrine development. Excessive stress that may challenge or perturb the neuroendocrine system when it is still developing could potentially have far-reaching consequences. This way, early-life stress may alter pain, neuroimmune, and neuroendocrine responses for life (4, 65–69).

## Early Development of the Immune System

### Immaturity of the Neonatal Immune System and Susceptibility to Infection

Infant mortality due to infection is high particularly in developing countries with a high prevalence of infection during the neonatal period (70). This high susceptibility of neonates and preterm infants to infection is thought to be due to immaturity of the neonatal immune system. Analysis of umbilical cord from preterm infants revealed fewer naïve CD8^+^ T cells and regulatory CD31 expression compared to full-term neonates (71). T cells play an important role in the control of intracellular infections. Both human and murine neonates lack mucosally distributed memory CD8^+^ T cells. Although T cell and cytokine mRNA levels [i.e., interleukin (IL)-1β, IL-6, and IFN-δ] can be detected in the thymus of mice from GD15 (72), neonatal mouse macrophages do not react in an adequate way early in life. For example, T-cells are characterized by lower IFN-δ responses following stimulation (73, 74). *Ex vivo* stimulation with the bacterial mimetic LPS in mice produced much less pro-inflammatory and anti-inflammatory cytokines response in neonates compared to adult mice (75). The same trend was observed in a human study whereby neonatal monocytes and dendritic cells produced less tumor necrosis factor (TNF)-alpha, IL-12, and IL-6 following LPS stimulation (76). When stimulated with an anti-CD3 antibody, neonatal T cell proliferation significantly decreased compared to adult T cell proliferation. This attenuation of proliferation in neonatal T cells was restored to adult levels following the addition of exogenous IL-2 (77). Furthermore, the total cell number of T cell subtypes (CD4^+^, CD8^+^, and Thy1^+^) is markedly lower in the spleen and lymphoid nodes in P4 mice compared to adult mice (78). Similarly, the function of antigen-presenting cells (APCs) is markedly decreased in human and murine neonates compared to adults (78). Treatment of both immunocompetent and immunodeficient mice with IL-12, a cytokine produced by APCs (79), prior to inoculation with the parasite *Cryptosporidium parvum* oocysts markedly reduced the severity of infection (80). Additionally, neonatal mice exhibited reduced levels of peripheral IL-12, and mice treated with IL-12 24 h after birth displayed increased levels of IFN-δ and IL-10 mRNA in the spleen (81). Adult humans exhibited much higher levels of granzyme B^+^ effector differentiated memory CD8^+^ T cells, which are thought to be the first responders to infections (82), than human neonates (83).

The incidence of sepsis, defined as a systemic inflammatory condition that occurs following exposure to pathogenic microorganisms or their toxins, is more than 25 times higher in infants less than 1 year compared to children from 1 to 14 years of age and constitutes a major risk of mortality and morbidity in the pediatric population (84). The incidence of infections is particularly high during the first postnatal weeks and rapidly decreases thereafter (85). Common causes of infections in neonates include commensal bacteria such as *Escherichia coli* (85). Both adaptive and innate neonatal immune responses are relatively immature as indicated by a lack of preexisting memory and decreased Th1-type responses (86, 87) as well as impaired production of TNFα following exposure to LPS (88, 89). Neonatal monocyte dendritic cells (moDC) also showed decreased production of interferon-β (IFN-β) in response to *in vitro* stimulation with LPS compared to mature adult moDC (89, 90). Additionally, whole blood neutrophil concentrations in 1-month children are shown to be lower than those in adults (91).

This immaturity of the immune system during neonatal life may thus predispose the neonatal immune system to infection, both of intra- and extracellular types. Overall, bacterial infection is considered the number one cause of perinatal infection in newborns worldwide (92, 93), which results in increased infant mortality particularly in developing countries (92, 94). In the coming sections, we argue that this sensitivity of the immune system early in life may have long-lasting effects in the adult organism.

### Infection As a Perinatal Stressor

Exposure to pathogens early in life is a common event and is considered to play a crucial role in priming the neuroendocrine–neuroimmune interface (95). An infection may not only be life-threatening to an infant but may also reorganize the function of the nervous system, due to the tight interplay between the nervous and immune systems. Human and animal studies have demonstrated that perinatal exposure to an immune challenge can produce changes in the CNS structure and function, leading to an increased risk of developing behavioral and psychopathological alterations later in life (66, 96–100). For instance, offspring from mothers exposed to infections such as influenza, LPS, and viral RNA (Poly I:C) during pregnancy have higher risk of developing schizophrenia and autism (101–106). A significant number of human and animal studies have also indicated that perinatal infection can alter immune (97, 107–110), metabolic (111, 112), reproductive (113, 114), endocrine (95, 115, 116), neurological (117, 118), and cognitive and behavioral responses later in life (98, 119, 120). Interestingly, exposure to LPS in rodents and humans can also cause pain facilitation such as thermal hyperalgesia, mechanical allodynia, and hyperalgesia (121–125). Such behavioral findings appear to be the result of altered peripheral and central cytokine activity (122, 126–128). Increased levels of pro-inflammatory cytokines, including IL-1β, TNF-α, and IL-6 produced by the maternal or fetal immune system, have been linked to abnormal brain development and increased risk of developing psychopathology (96, 98–100). Moreover, higher amounts of IL-6 in the amniotic fluid following bacterial infection during pregnancy have been previously reported to strongly correlate with increased mortality rates and brain injury (129).

Taken together, these findings highlight the fundamental role of the microbial environment in programming behavioral and neural responses. In order to understand the mechanisms of perinatal neuroendocrine–neuroimmune interaction, researchers employ experimental models that mimic the antigenic actions of infection.

## LPS as an Experiemental Immunological Stressor

Lipopolysaccharide, a complex glycolipid that is the major component of Gram-negative cell wall usually derived from *Salmonella enteritidis* or *E. coli*, is a powerful activator of innate immune responses and induces behavioral symptomatology in the host largely identical to those induced by live bacterial infection (130, 131). LPS-induced inflammation model presents well-known advantages, the primary one being that LPS does not replicate, allowing tight control of dosage and limiting the confounding nature of infection as compared to live bacteria models. LPS is commonly used to understand the complexities of the neuroimmune–neuroendocrine relationship and has been demonstrated to be a reliable activator of innate immune responses (97, 108) and HPA axis (66, 95, 108, 116, 132). Thus, LPS acts as an experimental systemic immunological stressor (133).

Lipopolysaccharide activates toll-like receptors and initiates a cascade of signalization leading to cytokine production that is crucial for infection clearance (134). Monocytes, neutrophils, macrophages, dendritic cells, and mast cells all express TLR4 at their surface membrane (135–137). Upon activation of the TLR4/MD2 complex by LPS, a series of phosphorylation steps are activated, leading to the phosphorylation of inhibitory (I)κB, which releases nuclear factor (NF)-κB from its complex (138). NF-κB is subsequently translocated into the nucleus where it activates the transcription of pro-inflammatory cytokines such as IL-1β, TNFα, and IL-6, as well as anti-inflammatory cytokines such as IL-1 receptor antagonist (IL-1ra) and IL-10 (139, 140). Cytokines released in the blood stream are able to activate the release of cyclooxygenase (COX)-2 from the hypothalamus to induce hyperalgesia (141). COX-2 also stimulates the conversion of arachidonic acid into prostaglandins (PGE_2_), which acts in the vascular organ of the lamina terminalis and in the ventromedial preoptic area of the anterior hypothalamus to stimulate heat conservation *via* cutaneous vasoconstriction and attenuation of sweating, and heat production *via* increases in the metabolism of brown adipose tissue (142). Circulating IL-1β is also known to directly activate hypothalamic PVN to stimulate the release of corticosterone from adrenal cortex (143, 144). LPS activation of Kupffer cells in the liver is also known to activate the release of IL-1β that can contribute to hyperalgesia *via* vagal afferences (145), as vagotomy abolishes the LPS-induced hyperalgesia (145).

### Neonatal LPS Exposure Changes Immune Responses Later in Life

Several lines of evidence from clinical and animal work suggest that exposure to LPS during the neonatal period is associated with altered immune responses later in life (66, 97, 109, 146–150). Most importantly, long-term inflammatory responses within the CNS are greatly influenced by immunological stressors early in life. Incubation of cord blood from 1-month old children with LPS for 5 h resulted in increased mRNA expression of IL-6 and TNFα compared to cord blood from the same age incubated with medium (146). In rats, neonatal LPS exposure produces immediate upregulation of gene expression of chemokines and cytokines within the neonatal brain, as indicated by upregulation of mRNA levels of Ccl7, Cxcl1, Cxcl10, IL-1β, and IL-6 in the hippocampus 2 h following LPS exposure in rat pups at PND 4 (151). The effect of neonatal LPS exposure on cytokine levels in limbic areas can persist into adulthood. Our laboratory has previously shown that neonatal LPS exposure at PNDs 3 and 5 results in increased IL-1β and TNFα protein levels in the hippocampus following exposure to restraint stress in adulthood (66). Recent investigations point toward a critical role played by the hippocampus in modulating pain *via* upregulation of IL-1β expression (152). del Rey et al. documented a strong correlation between increased hippocampal IL-1β transcripts and mechanical allodynia in chronic constriction injury and spared nerve injury (SNI) models (152). However, it is not known whether changes in protein levels of IL-1β in the hippocampus contribute to increased pain sensitivity in inflammatory pain models (i.e., formalin test). Neonatal immune challenge has also been reported to alter febrile responses later in life (147, 148, 150). Fever is considered an important component of the innate immune response and is thought to play a crucial role in survival through its ability to efficiently clear the pathogen while limiting the extent of inflammatory damage (153, 154). Animals prevented from developing fever have higher risk of morbidity and mortality than animals that are allowed to develop fever (155). Rats exposed to LPS at P14 exhibited attenuated fever responses following a subsequent LPS challenge (147, 149) or stress (150) in adulthood. The effect of neonatal LPS exposure on adult febrile responses is thought to be mediated by pro-inflammatory cytokines, as neonatally LPS-treated rats displayed significantly reduced plasma levels of TNFα and IL-6 following subsequent LPS exposure in adulthood. This reduction in turn was strongly correlated with the observed attenuated febrile responses in LPS animals (147). Interestingly, basal maintenance of body temperature in adult rats was not affected by neonatal LPS administration (110). This finding implies that a single LPS exposure is not able to alter febrile responses later in life, but that a “second hit” is necessary to “unmask” the altered febrile responses following a neonatal immune challenge. Central levels of PGE_2_ and specifically in the preoptic region, a region involved in the febrigenic thermoeffector pathways (156, 157), have also been targeted as potential mechanisms mediating the attenuated febrile responses following a neonatal immune challenge. For instance, PGE_2_ levels in the preoptic area were increased in rats exposed to LPS at P14 (150). Additionally, glucocorticoids play a critical role in inducing the febrile response, as adrenalectomy or blockade of GRs using the GR antagonist RU-486 abolished the fever induced by neonatal exposure to LPS (147). Finally, our laboratory has previously demonstrated that rats exposed to LPS at PNDs 3 and 5 displayed increased susceptibility to tumor and lung metastases following exposure to stress in adulthood (97, 108). Moreover, neonatal immune challenge produced reduced NK cell activity and increased neuroendocrine responsivity to restraint stress in adulthood (97, 108).

Taken together, an early immunological stressor has profound effects on the immunological reaction pattern later in life, leading to altered neuroimmune function at subsequent exposures to immunological challenges. This implies that what the immune system of an organism has been exposed to very early in life will in fact define its capacity to defeat pathogens later in life.

### Impact of Neonatal LPS Exposure on Endocrine Function

Microbial microbiota can affect the postnatal development of HPA axis, and an increasing body of evidence has demonstrated that neonatal exposure to LPS is associated with long-term alterations in HPA axis activity (66, 97, 116, 149, 158). Neonatal exposure to LPS during P3 and 5 has been reported to increase circulating levels of corticosterone at both time points (66, 132, 159), suggesting that neonatal LPS exposure is capable of altering HPA axis function during the SHRP. This alteration in HPA axis function following a neonatal immune challenge persists throughout the life of the animal. Adult rats treated with LPS as neonates displayed enhanced plasma corticosterone and ACTH levels in response to restraint stress, noise stress, or in response to a second LPS hit in adulthood (66, 95, 97, 116, 132). This altered peripheral endocrine response was also accompanied by central neuroendocrine changes, as indicated by increased CRH mRNA levels in the PVN and decreased GR density in the hypothalamus, hippocampus, and frontal cortex following exposure to stress in adulthood (95). These structures are known to mediate the inhibitory effects of glucocorticoids on CRH synthesis in the PVN and the release of ACTH following stress (160, 161), suggesting a decreased negative feedback sensitivity to glucocorticoids and, thus, an enhanced HPA responsiveness to stress following a neonatal immune challenge. We have demonstrated in our laboratory that dual exposure to LPS during P3 and P5 in rats is associated with increased circulating corticosterone at P7 and P22, but not P13, 1 h following injection of formalin into the hind paw (68). P22 rats neonatally treated with LPS also exhibited a trend toward decreased GR mRNA in the hypothalamus (68).

Overall, these data suggest that exposure to LPS during the neonatal period can reprogram the neuroendocrine axis. This reprogramming increases the reactivity of animals to a second physiological challenge later in life. Pain is an aversive experience and, therefore, capable of activating the HPA axis (162). Given that neonatal LPS exposure has been associated with increased release of peripheral and central pro-inflammatory cytokines later in life (66, 151) and considering the well-established role of pro-inflammatory cytokines in producing hyperalgesia (145), it is reasonable to assume that neonatal LPS exposure is likely to be associated with increased pain sensitivity later in life.

### Impact of Neonatal Exposure to LPS on Nociceptive Responses

The first postnatal week (P7–P10) of rodent’s life is equivalent to the last trimester in humans (36–40 GW) in terms of brain growth, gliogenesis, axonal and dendritic density, as well as consolidation of the immune system (11, 163–165). Preterm infants are, as discussed earlier, at high risk of infection during the neonatal period. Early-life infections in turn are known to be the cause of attenuated neurodevelopmental outcomes in these vulnerable infants (166). It is, therefore, important to address the impact of immune challenge on pain sensitivity later in life. Boisse et al found that administration of LPS at P14 in rats produced thermal and mechanical hyperalgesia that paralleled the enhanced expression of COX-2 protein levels in the lumbar spinal cord (141). Although this study did not directly demonstrate that the increased level of COX in the spinal cord contributed to the observed hyperalgesia in LPS-treated animals, it suggested a potential role of prostaglandins in mediating the LPS-induced hyperalgesia. Increased COX mRNA levels were also observed 4 h following LPS injection in P3 and P21 rats (P0 is birth) (167). A number of studies from our laboratory have indicated that dual exposure of LPS during P3 and 5 in rats produced long-term alterations in inflammatory pain responses later in life. Neonatal LPS administration evoked increased formalin-induced behavioral responses (i.e., flinching and licking) in P13, 22, and adult rats (4, 68, 168). The LPS-induced hyperalgesia observed in P22 rats coincided with altered HPA axis activity, as indicated by increased circulating corticosterone and decreased GR hypothalamic mRNA 1 h postformalin injection, as well as altered immune responses following formalin injection as indicated by increased mast cell degranulation and increased circulating IL-1β (4, 68). Moreover, the LPS-induced hyperalgesia in preadolescent rats was accompanied by altered spinal dorsal horn (SDH) intrinsic properties, as well as decreased neuronal activity (i.e., Fos expression) in the PAG (68, 168). LPS-treated adult rats exhibited hyperalgesia that coincided with central neuroimmune changes, as indicated by increased IL-1β in the hippocampus 1 h postformalin injection. No differences were observed in peripheral IL-1β release or mast cell degranulation (4). Although we reported enhanced hippocampal ILβ in LPS-treated adult rats, we do not know which immune cell releases this pro-inflammatory cytokine following neonatal immune challenge and subsequent inflammatory challenge. Of particular interest, hippocampal parenchyma astrocytes have been recently shown to produce the cytokine CCL2 24 h post-LPS injection in adult mice (169), suggesting an important role of astrocytes in the neuroinflammation produced by systemic LPS injection.

Taken together, these data challenge the traditional concept that pain is originating solely from activation of neurons and suggest that components of the immune system play an imminent role in modulating pain sensitivity. Using LPS as a model of infection, LPS-induced hyperalgesia arises by both peripheral and central mechanisms. Peripherally, LPS triggers, e.g., macrophages to release pro-inflammatory cytokines that sensitize nociceptors (145, 170, 171). In fact, LPS can directly activate TRPA1-expressing neurons independent of TLR4 (172). Centrally, LPS can activate microglial cells in the spinal cord and astrocytes in brain regions such as the hippocampus and produce hyperalgesia (169, 173).

## The Neuroimmune Interface in Pain in the Adult Organism

As discussed so far, the exposure to immunological stressors very early in development of an individual has far-reaching effects on neural structure and function as well as on the immune and HPA axis activity. We have also pointed to defining changes for the adult pain system. In the mature body, the systems are fully developed and less malleable. However, the immune system continues to affect the function of the nervous system in a manner that drives pain sensitivity, by inducing functional changes. In this section, we describe some acute neuroimmune interactions in pain perception. Such neuroimmune interaction may potentially be of importance for the transition from acute to chronic pain in a long-term perspective.

### Animal Studies Demonstrate Inflammation-Induced Pain Sensitivity

The role of the immune system was traditionally viewed as protecting the organism from invading pathogens. However, it is now well established that the bidirectional interaction between the immune and nervous systems plays a crucial role in pain modulation (125, 174–177). Pro-inflammatory cytokines play an important role in this immune to brain bidirectional interaction (121, 145). When exposed to LPS, immune cells such as macrophages, monocytes, and mast cells release many pro-inflammatory cytokines such as IL-1β, TNF-α, and IL-6 into the circulation creating an “inflammatory soup” condition that enhances pain sensitivity by sensitizing nociceptors (178–180). These pro-inflammatory cytokines also signal to the brain to induce a set of physiological responses including fever, lethargy, decreased social interaction, decreased sexual activity, and decreased food and water intake, increased circulating corticosterone, collectively known as sickness behavior (181–183). Importantly, pain facilitation or hyperalgesia is considered to be an integral part of sickness behavior (121, 125). Peripheral inflammation can lead to central neuroinflammation *via* many different ways. First, through vagal afferences since subdiaphragmatic vagotomy reversed the hyperalgesia induced by IL-1β or LPS (145). Alternatively, cytokines can access the brain through areas that lack the blood–brain barrier (BBB) such as the *organum vasculosum lamina terminalis* (184). LPS produces IL-1β in the brain, which is initially restricted to choroid plexus and circumventricular organs, then diffuse to the brain side of BBB (185). Cytokines have also been suggested to enter the brain *via* active transport systems across the BBB (186, 187).

The first report on the impact of LPS exposure on pain responses was the study by Mason, who demonstrated that i.p. administration of LPS in adult rats significantly decreased tail flick latency, an effect that peaked at 1 h post-LPS administration (123). The LPS-induced thermal hyperalgesia observed in adult rats was reversed following the administration of IL-1ra (188), indicating that IL-1β is an important mediator of this hyperalgesia. Pro-inflammatory cytokines released by immune cells are known to induce hyperalgesia when administered both peripherally and centrally, particularly IL-1β (145, 170, 189, 190). For instance, intracerebroventricular (ICV) administration of the recombinant human IL-1β (rhIL-1β) in rats induced thermal hyperalgesia (170), while ICV injection of the IL-1β antagonist IL-1ra abolished this hyperalgesia (170). Intraplantar injection of IL-1β has been associated with increased discharge of SDH neurons in response to non-noxious stimuli (190). Local administration of IL-1ra decreased the LPS-induced hyperalgesia (171).

Interleukin-1β is also known to contribute to flinching responses in the formalin test given that an intraplantar injection in rats of antisera anti-IL-1β prior to formalin injection significantly attenuated flinching responses in the formalin test (191). We have previously shown that rats exposed to LPS during the neonatal period displayed increased circulating IL-1β at P22 in response to formalin injection (4). Adult rats previously subjected to neonatal immune challenge also displayed enhanced hippocampal IL-1β that coincides with the LPS-induced hyperalgesia at this age (4). The source of this hippocampal IL-1β is not known, but it is highly probable that it is originating from astrocytes or microglial cells within the hippocampus. Interestingly, at the same age (i.e., PND 22), and at the same time point following formalin injection (i.e., 1 h postformalin injection), we observed altered intrinsic properties of SDH, lamina I, and lamina II neurons in LPS-treated rats as indicated by lower input resistance compared to saline-treated rats (68).

Spinal dorsal horn neurons are the first component of the CNS to receive incoming noxious sensory information, and their output is determined by a combination of their synaptic inputs and intrinsic neuronal properties (192). Formalin injection is known to activate peripheral nerves, which results in turn in activation of dorsal horn neurons (193–195). Hind paw injection of formalin is associated with the release of numerous substances in the spinal cord, including prostaglandin E2 (196). Bath application of prostaglandin E2 results in changes in intrinsic properties of dorsal horn neurons including decreased input resistance (197). Since this change was only observed in LPS-treated preadolescent rats, it is possible that the neonatal exposure to LPS resulted in either an increase in pro-inflammatory cytokines within the spinal cord or an increased susceptibility of SDH neurons to pro-inflammatory cytokines. This assumption is confirmed by the fact that intrathecal administration of IL-1ra has been reported to block formalin-induced hyperalgesia (198). The source of spinal hyperalgesia seems to involve microglia and astrocytes since intrathecal administration of fluorocitrate, an inhibitor of glial metabolic function, blocked the formalin-induced hyperalgesia (198).

Additionally, IL-1β has been documented to act supraspinally to induce hyperalgesia. For instance, microinjection of IL-1β into the preoptic area of the hypothalamus is sufficient to induce thermal hyperalgesia (199). Of particular interest is the observation that IP or ICV administration of IL-1β has been documented to produce an increase in plasma levels of corticosterone and ACTH, an action that is mediated by the release of CRH from the PVN (144, 200). The neonatal immune challenge is likely to influence the generation of new neurons in the hippocampus. This assumption is confirmed by the fact that an intraplantar injection of the nociceptive inflammatory agent Complete Freund’s Adjuvant at P8 results in more BrdU and doublecortin-labeled cells, both measures of newborn neurons, in the SGZ of the dentate gyrus (201). Whether such neurons release IL-1β in response to neonatal LPS exposure remains to be determined.

At the peripheral level, the enhanced IL-1β plasma levels observed at PND 22 in LPS-treated rats coincide with higher degree of mast cell degranulation, which was also accompanied by increased formalin-induced nociception (4). Mast cells are located in the vicinity of primary nociceptive neurons and vasculature and their degranulation has been reported to regulate the excitability of nociceptive nerve endings (202). Mast cell degranulation can also produce thermal hyperalgesia *via* the production of nerve growth factor (203). Previous studies have documented an important role of mast cells in formalin-induced nociception. Blocking mast cell activity using the mast cell stabilizer cromolyn abolished formalin-induced pain responses in the late phase (204). Interestingly, mast cells are also known to express receptor for IL-1β and to produce IL-1β following inflammation (205).

### Inflammation-Induced Pain Sensitivity in Humans

The human physiology is much more sensitive to LPS provocation than that of rodents. To avoid the risk of sepsis, very low doses of LPS are used in humans (usually 0.2–4.0 ng/kg), the highest doses often requiring additional antipyretic pharmacological treatment. The most common dose for psychological research is around 0.4–1 ng/kg LPS from *E. coli*, which induces a clear rise of pro-inflammatory cytokines TNFα, IL-1β, IL-6, and IL-8 in the blood (206–208). Human studies can also benefit from vaccinations of healthy individuals as an inflammatory model, and patients undergoing immunotherapy can be studied. The behavioral outcomes of experimental immune activation are very similar to sickness behavior exhibited by experimental animals; individuals report increased anxiety, worsened mood, and increased pain sensitivity (205, 209, 210). Appetite is reduced, and fatigue and anhedonia increase parallel to decreased social interest (126). The immune activation also disrupts memory and cognition and changes motivation (6, 211, 212). In human studies with the lowest LPS doses, the effects can in fact be so subtle that blinding can be maintained.

#### Pain Sensitivity during Immune Provocation

So far, only LPS stimulations have been used to study the pain system specifically in humans, and several studies have shown that experimental immune activation increases pain sensitivity in humans, too. Deep (muscular and visceral) pain is more readily affected than superficial (cutaneous and mechanical) pain (207, 213, 214). Also, the change in pain sensitivity usually correlates with peripheral cytokine levels. As in all experimental pain research, the mode of pain stimulation as well as the pain intensity applied may affect the outcome. Threshold pain is not processed exactly the same way as suprathreshold (intense) pain, and pain from within the body is relayed to the brain in pathways partly distinct from those used to relay cutaneous pain (215). Also, the nociceptive effect may depend on the immunological pressure, i.e., the LPS dose in experimental models. Two studies show that threshold pressure pain sensitivity is affected the same way in men and women, despite the generally higher cytokine levels found in women during LPS stimulation (207, 216). Interestingly, no sex differences in psychological outcomes, such as anxiety or perceived health, are seen either despite the sex differences in cytokine release (207, 208). One study has, however, shown that women are indeed more affected by inflammation with regard to pain perception (207). In this study, the descending pain inhibition of women was weakened during LPS stimulation, while men remained unaffected. In parallel, women were more pain sensitive to intense cutaneous pain, too, while men only changed their perception of deep pain. Furthermore, one study using a high LPS dose (2.0 ng/kg) has in fact shown increased pain sensitivity to intense cutaneous pain in men. Sex differences in inflammation-induced pain sensitivity need further exploration. An intriguing mechanism for a potential sex difference was recently suggested in a murine study (217), where female mice did not require microglia activation to develop pain hypersensitivity, but appeared to have alternative routes *via* the adaptive immune system. This alternative route did not seem accessible to males. Future research will have to establish if these mechanisms are relevant for humans as well and their role in immune-driven pain sensitivity. Furthermore, sex-dependent alterations in neuroendocrine function in human subjects following LPS provocation have been shown (218). Healthy humans exhibited enhanced circulating levels of cortisol (peak response at 5 h post-LPS injection) after LPS injection (208, 219). The effect appears to be more pronounced in women (208), but the data are inconclusive (219). On a final note, experimental pain is sensitive to stress, which could potentially be a confounder in LPS studies on pain. Perhaps, surprisingly, however, stress levels generally remain low among the participants throughout the studies (207). Our experience is that because LPS stimulations, due to ethical considerations using bacterial endotoxin injections in healthy subjects, require very clear participant information and a hospital environment with experienced personnel and constant supervision, participants describe a feeling of safety and control even at higher, quite uncomfortable doses (such as 2.0 ng/kg).

#### Brain Activity during Experimental Immune Activation

Although the cytokines released during immune activation may affect and sensitize peripheral nerve endings, the main effect by which the immune system changes the function of the nervous system during sickness is believed to occur centrally *via* induced sickness behavior. It is reasonable to assume that changes in the emotional circuitries underlying the increased anxiety and depressed mood seen during immune activation may also lead to increased pain sensitivity due to overlapping function with the medial (affective) pain network (215), such as the amygdala, the cingulate, and prefrontal cortices. Also, as sickness is per definition an interoceptive signal, i.e., a signal of the internal state of the body (220), areas involved in interoception and homeostasis such as the insular cortex, which is also part of the pain network, could potentially be affected. Several studies have attempted to elucidate the neural correlates of sickness behavior in the human brain. Most studies have used functional magnetic resonance imaging (fMRI) with cognitive and emotional paradigms. The main methodological limitation for this type of research is the fact that only the lower LPS doses used in humans are compatible with a brain scanning protocol, i.e., those that do not induce nausea or shivering.

Only two studies have explored pain perception directly during brain imaging so far, one using visceral pain stimuli (deep pain measurement) and mechanical pinprick pain (cutaneous pain measurement) (221) and the other using pressure pain (deep pain) (222). Benson et al. (221) showed increased activation within the posterior insula, dorsolateral PFC, anterior midcingulate, and somatosensory cortices for visceral pain stimulation, but not mechanical pain provocation. These areas are involved in pain and affective processing, interoception, and homeostatic regulation. Karshikoff et al. (222) described decreased activity after LPS injection in the lateral PFC and rostral anterior cingulate cortex (ACC), areas involved in descending pain inhibition, which may point to an increase in inflammation-induced pain sensitivity *via* diminished endogenous pain regulation. Additionally, the LPS group showed increased pain-dependent activity in the anterior insular cortex compared to placebo.

Emotional and cognitive fMRI paradigms corroborate the involvement of the cingulate, insula, and prefrontal cortices when the brain adapts to immune activation (221, 223–227), which are core areas in affective pain processing and pain regulation. Using a vaccination protocol as experimental immune provocation, Harrison et al. have shown increased activity in the subgenual ACC during emotional stimuli and in areas involved in interoceptive function during a Stoop task, such as the brain stem, the cingulate, and anterior insula (225, 226). To maintain the same level of performance during peripheral inflammatory activity, regions of the PFC appear to be required (224, 225)—areas implicated in pain regulation and processing of affective components of pain. In several studies, the increased BOLD activity in these areas correlates with peripheral cytokine levels (210, 222, 226, 228, 229).

Immune challenge affects the levels of neurotransmitters in the brain (6, 230). The expression of sickness behavior can potentially be manipulated by drugs affecting neurotransmitter levels such as serotonin reuptake inhibitors, which are compounds often used to ameliorate chronic pain. Hannestad et al. (231) have, for example, shown that the effects on fatigue are ameliorated by pretreatment of serotonin reuptake inhibitors, but not by dopamine and noradrenaline reuptake inhibitor. Peripherally induced inflammation has also been shown to activate microglia directly (232, 233). This is of special importance for chronic pain, as microglia have been implicated in the establishment of chronic pain (121).

In the past decade, it has thus been shown that acute inflammation induces pain sensitivity in humans as well. Most importantly, acute inflammation has a global effect on brain function, modulating the neural function in several brain areas involved in pain perception. Although the experimental models used are of an acute character, similar mechanisms are likely to be involved when the organism is subdued to long-term inflammatory activity.

## The HPA System and Pain in Adult Organisms

Pain is not only modulated by immunological stressors but also by activation of the HPA axis. Pain is a sensory as well as an emotional experience. It is by nature a stressful event and, therefore, capable of activating the HPA axis. As we have mentioned, there is a large individual variability in developing chronic pain. One possible mechanism that may account for this individual variability in pain responses is how each individual responds to stressful events. Exaggeration or maladaptive response following stress may lead to altered pain responses. The HPA axis involves a defined neural circuit that comprises many brain regions including the amygdala, the mPFC, and the hippocampus. These areas are also important in pain modulation (234–237). In other words, a non-painful stressful stimulus is able to recruit parts of the same neural network involved in the pain response. Therefore, under conditions of stress, pain sensitivity may be exaggerated. Indeed, activation of CRH receptors in the amygdala facilitated pain responses through increased excitatory postsynaptic current in the parabrachio-amygdaloid synapse in rodents (238). Furthermore, administration of CRH into the CeA increased visceral nociception, as indicated by exaggerated number of abdominal muscle contractions in response to colorectal distension (239). On the other hand, the contribution of acute stress in analgesia commonly known as “stress-induced analgesia” has been traditionally well documented (240, 241), and at this point in time the exact contribution of cortisol in modulating pain is still a matter of debate within the scientific community.

In human clinical samples, some researchers have found that low back pain and enhanced musculoskeletal pain are often associated with hypocortisolemia (242, 243), while others demonstrated that patients suffering from chronic back pain displayed higher levels of cortisol compared to control group (244). This hypercortisolemia was associated with smaller hippocampal volume and higher pain-evoked response in the anterior parahippocampal gyrus (244). This variability in cortisolemia in pain condition not only may be due to the intensity of the stress response (245) but may also well depend on the neural circuit recruited following the stress stimulus, as the neural circuits within PVN are quite complex, and the final outcome depends on the nature of the stressor [for review, please see Ref. (237)]. In inflammatory pain model, such as the formalin test in rodents, LPS-induced hyperalgesia in infant and preadolescent rats coincided with increased circulating corticosterone 1 h following intraplantar injection of formalin (68). However, a recent study demonstrated that elevated levels of plasma corticosterone produced analgesia *via* attenuated C fiber-mediated spinal responses (246).

Overall, the abovementioned animal and human studies suggest that changes in HPA axis activity can contribute to pain. Although more studies are needed to confirm the exact contribution of cortisol (in humans) or corticosterone (in rodents) in modulating pain responses, the involvement of neuroendocrine response in pain is evident. Therefore, new therapeutic approaches, which not only target neural activity but also the neuroendocrine axis, are needed to treat chronic pain patients.

## A Lifetime Perspective

Although the acute effects of immune provocation on pain sensitivity are fairly well documented by now, as described in the previous sections, long-term inflammatory effects are not well understood. At this point in time, the most research on long-term effects of inflammatory activity on behavior has focused on depression. In humans, one incentive to study the mechanisms of sickness behavior came from clinical observations of immunotherapy eliciting side effects that resemble sickness behavior, such as depressive symptoms, fatigue, and aches. In, for example, hepatitis C patients undergoing IFN-α therapy, up to 45% of the patients develop depression (247). The typical signs of sickness behavior appear at the commencement of immunotherapy, whereas the establishment of depression requires time, and potentially, persistent inflammatory input during this time. It is now argued that depression is in part an inflammatory disease (248), and that a subgroup of clinically depressed patients suffers from a chronic low-grade systemic inflammation. Childhood trauma has also been shown to predispose persons to depression, but potentially not only *via* learning and HPA dysregulation as traditionally suggested but also *via* inflammation. Depressed patients with a history of traumatic events have higher low-grade inflammatory activity (249). Most interestingly, these patients benefit from pharmacological treatments that combine anti-inflammatory compounds and traditional antidepressants (249). Suggested mechanisms between inflammatory activity and depression include cytokines, serotonin, HPA dysregulation, GABA, and glutamate, all of which are neuroimmune pathways also implicated in pain [for extensive reviews see, e.g., Ref. (6, 250)]. Recent research is now shifting the focus toward similar mechanisms for chronic pain and fatigue (6, 230).

### Inflammatory Disease and Pain

Chronic pain is a common comorbid symptom to many inflammatory diseases (251). Moreover, coronary heart disease (252), metabolic disorders (253), and life stress (254) increase the risk of developing chronic pain. It has been suggested that one of the underlying mechanisms for this association is indeed inflammation (252–254). Furthermore, chronic pain has been associated with low-grade inflammation (255). Mechanistically, peripheral chronic inflammation may become chronic within the CNS *via* changes in the central immune responses, by means of mechanism previously discussed. In animals, transient peripheral infections and inflammations or chronic exposure to low level (subclinical) inflammations can either activate microglia directly (256, 257) or “prime” the cells so that a recurrent inflammatory provocation becomes more severe (258). A systemic inflammatory challenge leads to an exaggerated fever response and sickness behavior in the presence of “primed” microglia in rodents (259, 260). “Priming” of immune components, or the requirement of a “second immunological hit” to reveal susceptibility as discussed previously, is exemplified in a recent clinical study. Obesity has been associated with chronic pain and is considered a chronic low-grade inflammatory state (253). Obesity did not predict postsurgical pain intensity or inflammatory levels (255). BMI did, however, correlate with the increased immune response of leukocytes after LPS stimulation, suggesting sensitivity to inflammatory development in the obese patients that could results in complications associated with inflammation further down the road, such as chronic pain. Another study points to differences in pharmacological treatment strategies on pain after surgery, depending on prior inflammatory disease. Non-steroidal anti-inflammatory drugs had a better protective effect against the development of long-term pain after surgery in patients with a background of inflammatory disease, than opioids (261).

### The Immune System Develops throughout Life

As discussed previously, the immune system carries the imprint of early-life inflammatory events. However, the function of immune system in fighting previously unencountered pathogens and protect the organism on reinfection relies on the ability to adapt and learn throughout life. Immune functioning is determined partly by genetics (262) and varies greatly between individuals. Individuals differ in their susceptibility to different types of infections, such as bacterial, viral, and fungal (262), and several single nucleotide polymorphisms related to immune pathways have been described (262). For example, the IL-6 and IL-8 pathways appear to have large genetic variations between individuals, while the IL-1 pathway has remained more conserved throughout evolution (262). Recent research emphasizes the importance of experience in shaping the adult immune response, similar to what has been described for infants in the previous sections. In fact, most of the individual differences seen in immune function in adult humans stem from non-heritable changes (263, 264). The immune system activates distinct cytokine patterns depending on the type of infection, and continuously learns from experience to adapt its inflammatory response (265). In theory, each person thus possesses an immune system that is a product of the types, strengths, and number of infections, diseases, and injuries encountered throughout life. Prior experience should thus impact future immunological reaction patterns. Epidemiological studies on comorbidity and risk factors for common disease give support to the idea that lifetime immune challenges affect disease susceptibility. A recent study shows that in patients with multimorbidity (in this specific study more than 10 disease diagnoses), the incidence of lifetime infections, inflammation, injuries, and tumors was 7–10 times as common as in a primary health care population (266). Lifetime accumulation of strong immune activation may thus potentially lead to increased general disease susceptibility and comorbidity (266). Furthermore, lifetime inflammatory disease is a risk factor for developing neurodegenerative disease (267–269). A plausible mechanism is that the accumulation of inflammatory activity in the body induces neuroinflammation in the brain, which in turn affects the function of the CNS (267).

### When Adaptation Becomes a Liability

The process of perinatal programming posits that exposure to environmental factors during a sensitive window of development is able to program or have long-term consequences on physiological systems later in life. A fundamental aspect of perinatal programming is that developing organisms “sense” the early-life environment and use this information to establish homeostatic set points (270, 271). This process of perinatal programming has evolved as an adaptive mechanism enabling the fetus to constantly interact with the maternal environment (*via* the placenta) and use this information as a forecast of the environmental conditions it will eventually face postnatally. As such, preparing it to adjust its physiological and behavioral need to match the requirements of the *ex utero* world (272). In this perspective, fetal programming is an example of predictive adaptive responses where the fetus uses present cues to shape an adaptive phenotype to future environmental stimuli (31, 273). However, this adjustment can become maladaptive in the case where a “mismatch” exists between the expected *ex utero* environment and the actual circumstances. More importantly, when adverse events occur during a critical window of vulnerability of physiological systems that are still undergoing fine-tuning and plasticity, an individual may become predisposed to high susceptibility and exaggerated sensitivity to environmental stimuli later in life.

Correspondingly, the immune system and the HPA system adapt and change according to the stressors that the individual encounters throughout life, in order to maintain health and homeostasis. Pain is one of the most important survival signals available to us, and a life without pain perception is often a short one, as can be seen in individuals with congenital insensitivity to pain (274). However, when the imprint of the different stressors throughout life accumulate, interact, and/or become prolonged, the consequence may be detrimental for the pain system. For diseases like chronic pain, with such wide individual variability in symptomatology and treatment efficacy (8, 9), not only should comorbid disease and stressful life events (i.e., concurrent with the pain) be considered when exploring the pathophysiology but also past stressors. In this study, we want to increase the awareness of the profound effect of the immune system on the pain system from birth to old age, *via* neuroimmune and neuroendocrine interactions. In other words, the faith of the pain system starts *in utero*.

## Conclusion

In this review, we argue that the individual differences in the susceptibility to chronic pain and success of treatment thereof may be the result of the person’s prenatal history, combined with childhood as well as lifetime experience. We have highlighted the biological underpinnings and potential consequences on the pain system induced by the stress and infectious/inflammatory load an individual is subjected to. The neuroimmune and neuroendocrine interactions that affect the pain system start in the womb and modulate the pain system throughout life. The modulations may be of both structural and functional nature and may be both adaptive and maladaptive. In order to understand individual differences in pain, human studies of long-term effects of inflammatory stressors are needed.

## Author Contributions

IZ and BK wrote the manuscript and approved the final version.

## Conflict of Interest Statement

The authors declare that the research was conducted in the absence of any commercial or financial relationships that could be construed as a potential conflict of interest. The reviewer AF-H declared a shared affiliation, though no other collaboration, with the authors to the handling Editor, who ensured that the process nevertheless met the standards of a fair and objective review.
